# Performance-based incentives and community health workers’ outputs, a systematic review

**DOI:** 10.2471/BLT.20.285218

**Published:** 2021-08-20

**Authors:** Thomas Gadsden, Sikhumbuzo A Mabunda, Anna Palagyi, Asri Maharani, Sujarwoto Sujarwoto, Michelle Baddeley, Stephen Jan

**Affiliations:** a The George Institute for Global Health, University of New South Wales, Sydney, Level 5/1 King St, Newtown 2042, New South Wales, Australia.; b Faculty of Biology, Medicine and Health, The University of Manchester, Manchester, England.; c Department of Public Administration, University of Brawijaya, Malang, Indonesia.; d UTS Business School, University of Technology Sydney, Sydney, Australia.

## Abstract

**Objective:**

To review the evidence on the impact on measurable outcomes of performance-based incentives for community health workers (CHWs) in low- and middle-income countries.

**Methods:**

We conducted a systematic review of intervention studies published before November 2020 that evaluated the impact of financial and non-financial performance-based incentives for CHWs. Outcomes included patient health indicators; quality, utilization or delivery of health-care services; and CHW motivation or satisfaction. We assessed risk of bias for all included studies using the Cochrane tool. We based our narrative synthesis on a framework for measuring the performance of CHW programmes, comprising inputs, processes, performance outputs and health outcomes.

**Findings:**

Two reviewers screened 2811 records; we included 12 studies, 11 of which were randomized controlled trials and one a non-randomized trial. We found that non-financial, publicly displayed recognition of CHWs’ efforts was effective in improved service delivery outcomes. While large financial incentives were more effective than small ones in bringing about improved performance, they often resulted in the reallocation of effort away from other, non-incentivized tasks. We found no studies that tested a combined package of financial and non-financial incentives. The rationale for the design of performance-based incentives or explanation of how incentives interacted with contextual factors were rarely reported.

**Conclusion:**

Financial performance-based incentives alone can improve CHW service delivery outcomes, but at the risk of unincentivized tasks being neglected. As calls to professionalize CHW programmes gain momentum, research that explores the interactions among different forms of incentives, context and sustainability is needed.

## Résumé

**Objectif:**

Examiner les preuves attestant de l'impact des incitants liés aux performances sur les résultats mesurables des agents de santé communautaires (ASC) dans les pays à faible et moyen revenu.

**Méthodes:**

Nous avons effectué une revue systématique des études d'intervention publiées avant novembre 2020 et évaluant l'impact, sur les ASC, des incitants financiers et non financiers liés aux performances. Les résultats observés concernaient notamment les indicateurs de santé des patients; la qualité, l'utilisation ou la fourniture de soins de santé; et le degré de motivation ou de satisfaction des ASC. Nous avons employé l'outil Cochrane afin de calculer le risque de biais pour toutes les études sélectionnées. Notre synthèse narrative repose sur un cadre mesurant les performances des programmes d'ASC, en ce compris les moyens disponibles, les processus et les résultats en termes de performances et de santé.

**Résultats:**

Deux réviseurs ont passé au crible 2811 documents; nous avons retenu 12 études, dont 11 essais randomisés contrôlés et un essai non randomisé. Nous avons constaté que le fait d'afficher ouvertement une reconnaissance non financière des efforts consentis par les ASC entraînait une amélioration des prestations de service. En outre, bien que l'efficacité des incitants financiers soit proportionnelle au montant octroyé lorsqu'il s'agissait d'obtenir de meilleures performances, cette évolution se faisait souvent aux dépens des tâches ne donnant pas droit à des mesures incitatives. Nous n'avons trouvé aucune étude ayant testé un ensemble d'incitants financiers et non financiers. La logique derrière la conception des incitants liés aux performances ou l'explication des interactions entre incitants et facteurs contextuels n'étaient que rarement communiqués.

**Conclusion:**

Les incitants financiers liés aux performances suffisent à améliorer les prestations des ASC, mais risquent de détourner l'attention des tâches ne faisant pas l'objet d'incitants. De plus en plus de voix s'élèvent pour réclamer une professionnalisation des programmes d'ASC; il devient donc essentiel de mener des recherches afin d'explorer les interactions entre les différentes formes d'incitants, le contexte et la pérennisation.

## Resumen

**Objetivo:**

Revisar la evidencia sobre el impacto en los resultados medibles de los incentivos basados en el rendimiento para el personal sanitario comunitario (PSC) en países de ingresos bajos y medios.

**Métodos:**

Se realizó una revisión sistemática de los estudios de intervención, publicados antes de noviembre de 2020, que evaluaban el impacto de los incentivos financieros y no financieros basados en el rendimiento para el PSC. Los resultados incluyeron indicadores de salud de los pacientes; calidad, utilización o prestación de servicios de atención sanitaria; y motivación o satisfacción del PSC. Se evaluó el riesgo de sesgo para todos los estudios incluidos mediante Cochrane. La síntesis narrativa se basó en un marco para medir el rendimiento de los programas de PSC, que comprende aportaciones, procesos, resultados de rendimiento y resultados de salud.

**Resultados:**

Dos revisores examinaron 2811 informes; se incluyeron 12 estudios, 11 de los cuales eran ensayos controlados aleatorios y uno no aleatorio. Se encontró que el reconocimiento no financiero, mostrado públicamente, de los esfuerzos del PSC fue efectivo para mejorar los resultados de la prestación de servicios. Si bien los incentivos financieros grandes fueron más eficaces que los pequeños para lograr un mejor rendimiento, a menudo dieron lugar a la reasignación de esfuerzos de otras tareas no incentivadas. No encontramos ningún estudio que probara un paquete combinado de incentivos financieros y no financieros. Rara vez se informó de la justificación del diseño de los incentivos basados en el rendimiento o de la explicación de cómo los incentivos interactuaban con los factores contextuales.

**Conclusión:**

Los incentivos financieros basados en el rendimiento por sí solos pueden mejorar los resultados de la prestación de servicios del PSC, pero con el riesgo de que se descuiden las tareas no incentivadas. A medida que los llamamientos a la profesionalización de los programas de PSC cobran impulso, es necesario realizar investigaciones que exploren las interacciones entre las diferentes formas de incentivos, el contexto y la sostenibilidad.

## ملخص

الغرض مراجعة الدليل على التأثير على النتائج القابلة للقياس للحوافز القائمة على الأداء للعاملين في الصحة المجتمعية (CHWs) في الدول منخفضة الدخل ومتوسطة الدخل.

الطريقة لقد قمنا بإجراء مراجعة منهجية لدراسات التدخل التي تم نشرها قبل نوفمبر/تشرين ثاني 2020، والتي قامت بتقييم تأثير الحوافز المالية وغير المالية القائمة على الأداء للعاملين في الصحة المجتمعية. تضمنت النتائج مؤشرات صحة المريض؛ جودة خدمات الرعاية الصحية أو استخدامها أو تقديمها؛ ودوافع أو رضا العاملين في الصحة المجتمعية. قمنا بتقييم خطر التحيز لكل الدراسات المشمولة باستخدام أداة كوكرين. اعتمدنا في تجميعنا السردي على إطار عمل لقياس أداء برامج العاملين في الصحة المجتمعية، والذي يشمل المدخلات، والعمليات، ومخرجات الأداء، والنتائج الصحية.

النتائج قام اثنان من المراجعين بفحص 2811 سجلاً؛ وتضمين 12 دراسة، 11 منها كانت تجارب معشاة ذات شواهد، وواحدة غير معشاة. ووجدنا أن التقدير العلني غير المالي لجهود العاملين في الصحة المجتمعية كان فعالًا في نتائج تقديم الخدمات المحسّنة. في حين أن الحوافز المالية الكبيرة كانت أكثر فعالية من الحوافز الصغيرة في تحسين الأداء، إلا أنها غالبًا ما أدت إلى إعادة تقسيم الجهود بعيدًا عن المهام الأخرى غير المحفزة. لم نعثر على دراسات قامت باختبار حزمة مشتركة من الحوافز المالية وغير المالية. نادرًا ما تم الإبلاغ عن الأساس المنطقي لتصميم الحوافز القائمة على الأداء، أو شرح كيفية تفاعل الحوافز مع العوامل السياقية.

الاستنتاج يمكن للحوافز المالية القائمة على الأداء وحدها أن تحسن من نتائج تقديم خدمات بواسطة العاملين في الصحة المجتمعية، ولكن مع خطر إهمال المهام غير المصحوبة بالحوافز. مع انتشار الدعوات لإضفاء الطابع الاحترافي على برامج العاملين في الصحة المجتمعية، فإن هناك حاجة لإجراء بحث يستكشف التفاعلات بين الأشكال المختلفة للحوافز والسياق والاستدامة.

## 摘要

目的 旨在回顾中低收入国家基于绩效的奖励措施对社区卫生工作者 (CHW) 可衡量成果的影响方面的证据。

方法 我们对 2020 年 11 月之前发布的干预性研究开展了系统性回顾，评估基于绩效的财务和非财务奖励措施对社区卫生工作者的影响。工作成果包括患者健康指标；医疗保健服务的质量、使用或交付；以及社区卫生工作者的积极性和满意度。我们采用 Cochrane 工具评估了所有囊括在内的研究的偏倚风险。我们基于一个衡量社区卫生工作者绩效表现的计划、要素输入、流程、绩效输出和健康成果的框架来形成我们的叙述性综论。

结果两位审核人筛选了 2811 条记录；我们采纳了 12 项研究，其中的 11 项研究为随机对照试验，一项为非随机试验。我们发现，非财务的公开表彰社区卫生工作者的贡献在提升服务成果方面具有成效。尽管高额财务奖励比小额奖励在提升绩效表现方面更有成效，但会导致社区卫生工作者将精力从其他无财务奖励的任务上转移。我们没有发现任何研究对包含财务奖励和非财务奖励的综合奖励措施进行过考察。报告中很少提到基于绩效的奖励措施设计原理或者关于奖励措施如何体现背景因素的解释。

结论单独采取基于绩效的财务奖励措施可以提高社区卫生工作者所提供的服务成果，但存在忽视无奖励任务的风险。随着呼吁专业化社区卫生工作者计划的声音越来越高，有必要开展研究以探索不同奖励形式、背景和可持续性之间的相互作用。

## Резюме

**Цель:**

Провести обзор данных о влиянии стимулов для общинных медико-санитарных работников (ОМСР), применяемых с учетом результатов их деятельности, на поддающиеся оценке исходы такой работы в странах с низким и средним уровнем доходов.

**Методы:**

Авторы провели систематический обзор интервенционных исследований, опубликованных до ноября 2020 года, в которых оценивалось влияние материальных и нематериальных стимулов для ОМСР с учетом результатов их деятельности. Конечные результаты включали в себя показатели здоровья пациентов; качество, использование или предоставление медицинских услуг; мотивацию или удовлетворенность ОМСР. Был оценен риск систематической ошибки для всех включенных исследований, для чего использовался инструмент Кокрановского сотрудничества. В своем нарративном синтезе авторы опирались на рамочную схему измерения эффективности программ для ОМСР, включая затрачиваемые ресурсы, процессы, показатели эффективности реализованных мероприятий и результаты мероприятий по охране здоровья.

**Результаты:**

Два рецензента просмотрели 2811 записей. В обзор вошли 12 исследований, 11 из которых были рандомизированными контролируемыми исследованиями, а одно — нерандомизированным исследованием. Мы обнаружили, что нематериальное, публичное поощрение усилий ОМСР является эффективным средством для улучшения результатов предоставления услуг. Хотя крупные материальные стимулы были более эффективными, чем мелкие, в плане повышения эффективности деятельности, они часто приводили к перераспределению усилий, которые в противном случае могли быть потрачены на решение других, нестимулируемых задач. Мы не нашли исследований, в которых испытывался бы комплексный пакет материальных и нематериальных стимулов. Об обосновании для разработки стимулов с учетом результатов деятельности или об объяснении того, как стимулы взаимодействуют с контекстуальными факторами, сообщалось редко.

**Вывод:**

Материальные стимулы с учетом результатов деятельности сами по себе могут улучшить результаты предоставления услуг ОМСР, но с риском того, что нестимулируемые задачи будут оставлены без внимания. Поскольку призывы к профессионализации программ для ОМСР набирают силу, необходимы исследования, изучающие взаимодействие между различными формами стимулов, контекстом и экологически и социально ответственным поведением.

## Introduction

Community health workers (CHWs) play an important role in fulfilling global commitments to strengthen primary health-care systems and achieve universal health coverage. A CHW is a member of the community who has received some training to promote health or to perform some health-care services but is not a health-care professional.^1^ They provide basic preventive health care, health education, referral and home visiting services to specific communities.^2^^,^^3^ Although CHWs have lower levels of education and training than other health workers such as doctors and nurses, they are directly connected to the communities they serve – they live in them and are accountable to them.^3^^,^^4^ Staffing models for CHW programmes worldwide range from highly trained salaried workers to volunteers with minimal training.^5^

Performance-based incentives refer to the transfer of money or material goods conditional on the achievement of a predetermined performance target.^6^ Incentives can include financial incentives such as commissions, stipends and allowances, as well as non-financial incentives such as material goods, certificates and awards.^7^ In low-and middle-income countries these interventions are typically implemented at the facility level but they can be directly targeted to individual health workers.^8^^,^^9^ Measurable targets may include health outcomes, delivery of interventions, utilization of services and quality of care.^8^^,^^9^While many published reviews highlight incentives as an important element of CHW programmes, we found no specific evidence on the impact of performance-based incentive interventions on measurable outcomes of CHWs’ performance.^4^^,^^10^^–^^12^ A systematic review was conducted to inform the 2018 World Health Organization (WHO) guideline to optimize CHW programmes.^3^^,^^13^ However, the study focused on the broader question of whether CHWs should be paid for their work. The impact of performance-based incentives on outcomes for other cadres of health workers has been widely studied, but similar evidence for CHWs is lacking.^14^^–^^16^

The design of performance-based incentives for health workers and the mechanisms by which incentives influence behaviour remains poorly understood.^6^ Introducing payments for tasks which were previously performed as part of the job or on a voluntary basis may undermine intrinsic motivations (that is, engaging in a behaviour because it is rewarding).^17^ Performance-based incentives may also detract effort from other unrewarded tasks.^18^ For instance, rewarding health workers for reaching performance targets (such as number of patients screened for cardiovascular risk factors) may reduce their motivation to conduct unrewarded activities (such as dietary and lifestyle advice). These considerations contributed to the 2018 WHO guideline recommendation that CHWs should not be paid “exclusively or predominantly according to performance-based incentives.”^13^ The guideline also cites insufficient context-specific evidence as a key research gap and that “evidence is not sufficiently granular to allow recommendation of specific forms of interventions, for example which bundle of financial and non-financial incentives are most effective.”^13^

We carried out a systematic review of the impact of performance-based incentives on measurable outcomes of CHWs’ performance. Our goal was to examine the design features that may explain why some interventions were more successful than others. This review comes at a time of heightened interest in designing behaviour change interventions through frameworks such as behavioural economics. Consolidating such evidence in relation to the behaviour of CHWs and their interactions with performance-based incentives will equip policy-makers to design more effective interventions.

## Methods

We designed the review in accordance with the Preferred Reporting Items for Systematic Reviews and Meta-Analyses guidelines and registered it prospectively with the International Prospective Register of Systematic Reviews (#CRD187629).^19^

### Study selection

We searched online databases for studies reporting the implementation of incentives to improve the performance or motivation of CHWs in low- and middle-income countries and published before November 2020 (Box 1).The full search strategy is available in the authors’ data repository.^20^

Box 1 **Databases and example search terms for the systematic review on incentives for community health workers in low- and middle-income countries**

We searched the online databases of PubMed®, Embase®, Emcare®, Global Health, Cochrane Library, PsycInfo® and CINAHL, using the following keywords:

(Community Health Work*/ OR lay health work*/OR volunteer health work*/ OR Home Health Aides)

AND

(Motivation/ OR reimbursement, incentive/ OR salaries and fringe benefits/ OR remuneration/ OR Performance based incentive/ OR Attitude of Health Personnel/ OR employee incentive plans/ OR gift giving/)

AND

Developing Countries/ OR ((low adj3 middle adj3 countr*) OR (lmic OR lmics OR lami countr* OR middle income countr*))

We used the definition of CHWs from the 2018 WHO guideline to optimize CHW programmes,^13^ which excludes traditional birth attendants.^3^ We limited eligibility to studies where one of the primary objectives was to determine the impact of an intervention of performance-based incentives on measurable outcomes of CHW performance. We included incentives that were financial or non-financial or a combination. To measure change in CHW performance, we extracted and categorized outcomes as: patient health; quality, utilization or delivery of health-care services; and CHW motivation or satisfaction. We included motivation as an outcome measure as it is an important component of performance.^21^ Eligible study designs included randomized and non-randomized trials, controlled before-and-after studies and impact evaluation studies. We excluded qualitative studies and studies where the impact of the incentive was not isolated from the overall impact of the CHW programme (for example in a study in Uganda).^22^ We limited the search to countries classified as low income and lower-middle income in the World Bank income classification in 2019.^23^ We applied no language restrictions.

Two authors independently assessed the titles and abstracts of the initial search results against the inclusion and exclusion criteria and reached a consensus. Full texts were then reviewed by the same two authors and where necessary a third reviewer was consulted to resolve disagreements. We hand-searched the reference lists of all included articles for additional articles.

### Data analysis

Two authors independently extracted the following data into a spreadsheet: study design, country, CHW characteristics and outcomes. We assessed risk of bias using the Cochrane risk-of-bias tool.^24^ Two authors independently assessed the quality of each included study using the Critical Appraisal Skills Programme quality checklist.^25^ We selected these tools for their ability to identify key quality issues across a wide range of study designs, including those incorporating complex interventions. More details of the quality criteria used are shown in the data repository.^20^

**Fig. 1:**
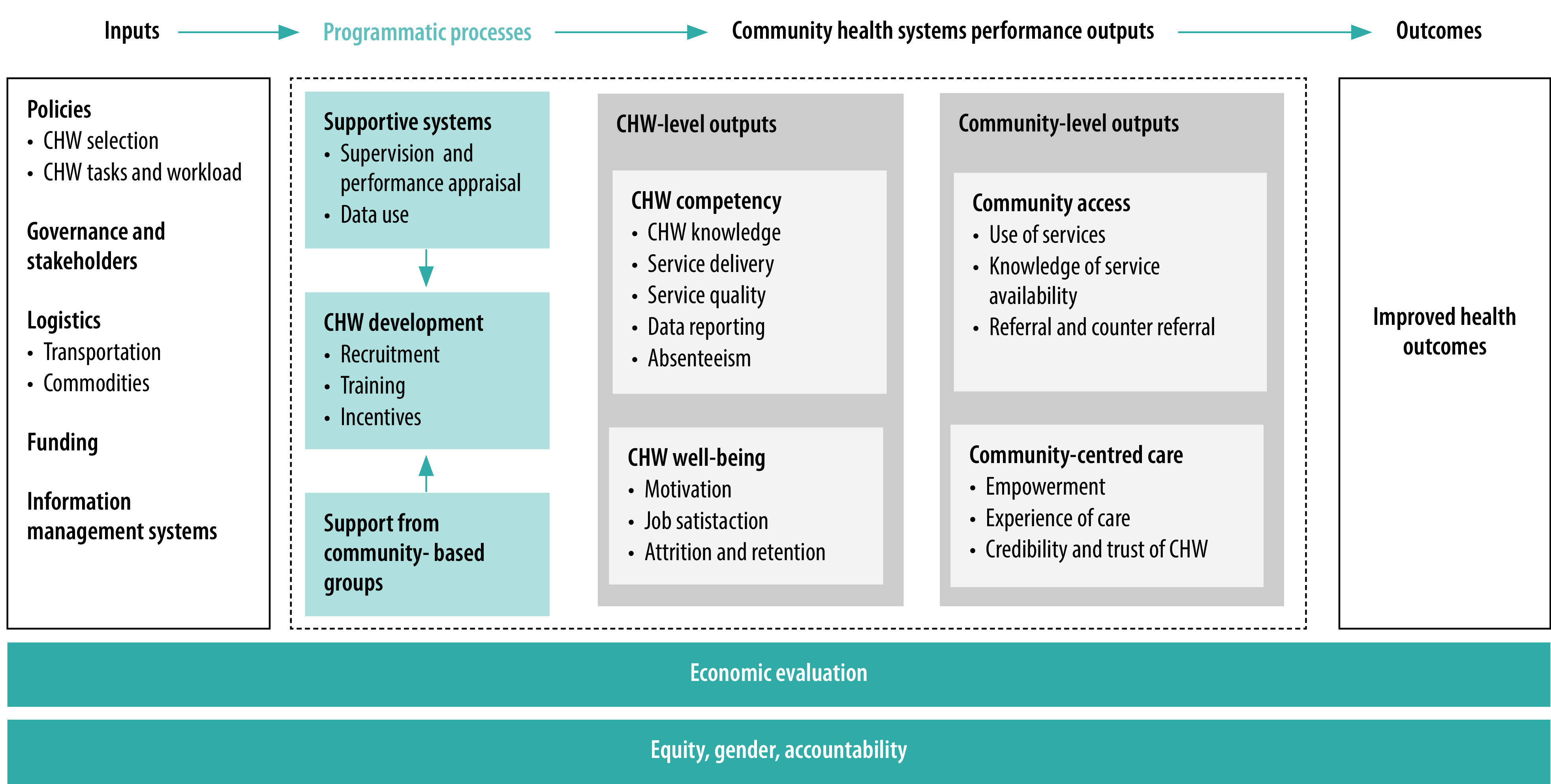
Community health worker performance measurement framework

We performed a narrative analysis that was informed by a previously published framework for measuring CHW performance (Fig. 1).^26^ The framework identifies domains for measuring the performance of CHW programmes, comprising: inputs, processes, performance outputs and outcomes. As we restricted our review to CHW performance outcomes, our synthesis focused on outputs and outcomes under the framework. Where possible, we also synthesized the results according to their pathways to impact. This technique reflects recent practice in the performance-based financing community to go beyond assessing the average effect of programmes to exploring the mechanisms of impact.^27^

CHW: community health worker.

Source: Agarwal et al., 2019.^26^ Right to reproduce under the Creative Commons Attribution 4.0 International License (http://creativecommons.org/licenses/by/4.0/).

## Results

**Fig. 2:**
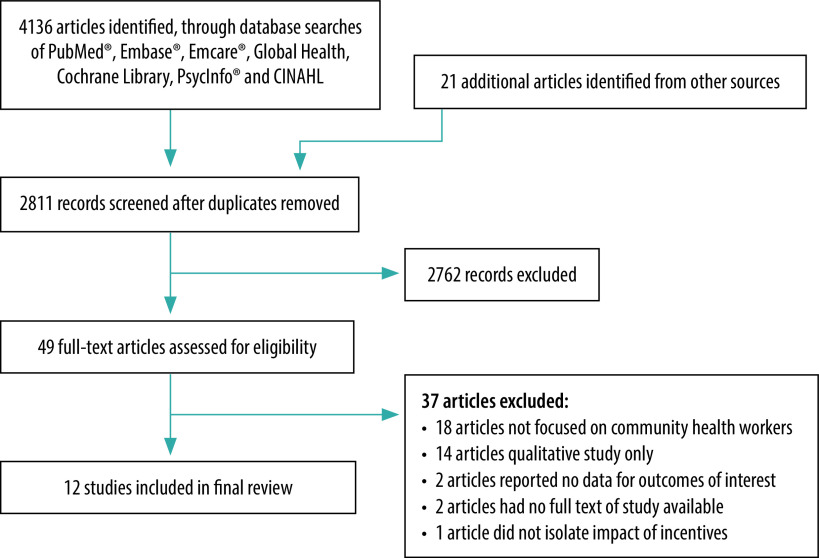
Flow diagram of selection of articles for the systematic review of performance-based incentives for community health workers

Our initial search yielded 2811 records, 49 of which met our inclusion criteria and underwent full-text review. We included 12 studies in the final review^28^^–^^39^ from eight countries (El Salvador, Guinea-Bissau, India, Pakistan, Rwanda, Sierra Leone, Uganda and Zambia; Fig. 2). The characteristics of the included studies are summarized in Table 1. The studies provided limited information on the baseline function of CHW participants, their baseline forms of remuneration and how incentives were designed (more details are in the data repository).^20^

**Table 1:** Studies included in the systematic review of performance-based incentives for community health workers in low- and middle-income countries

Study	Country	Setting	Study design	Study duration	Sample size (no., % women)	Type of incentive^a^
Ashraf et al., 2014^36^	Zambia	Urban	Four-arm cluster randomized controlled trial	12 months	771 community health agents (% of women not reported)	Financial and non-financial
Singh, 2015^31^	India	Urban slums	Non-randomized trial	3 months	145 Anganwadi workers (145 women, 100%)	Financial
Bossuroy et al., 2016^28^	India	Urban slums	Four-arm cluster randomized controlled trial	12 months	78 CHWs (78 women, 100%)	Financial
Singh & Masters, 2017^32^	India	Urban slums	Three-arm cluster randomized controlled trial	3 months	160 Anganwadi workers (160 women, 100%)	Financial
Singh & Mitra, 2017^33^	India	Urban slums	Three-arm cluster randomized controlled trial	3 months	209 Anganwadi workers (209 women, 100%)	Financial
Shapira et al., 2018^30^	Rwanda	Urban and rural	Four-arm randomized controlled trial	4 years	197 CHW cooperatives; 2000 CHWs (1720 women, 86%)	Financial
Carmichael et al., 2019^29^	India	Urban	Five-arm cluster randomized controlled trial	2.5 years	646 accredited social health activists, Anganwadi workers and auxiliary nurse midwives (646 women, 100%)	Non-financial
Fracchia et al., 2019^38^	Guinea-Bissau	Urban	Three-arm randomized controlled trial	14 months	1015 CHWs (487 women, 48%)	Non-financial
Bernal & Martinez, 2020^37^	El Salvador	Urban and rural	Cluster randomized controlled trial	18 months	75 community health teams (% of women not reported)	Non-financial
Wagner et al., 2020^34^	Uganda	Peri-urban	Cluster randomized controlled trial	1 month	118 CHWs (% of women not reported)	Financial
Deserranno et al., 2020^35^	Sierra Leone	Six districts	Four-arm randomized controlled trial	14 months	2970 CHWs (861 women, 29%)	Financial
Khan, 2020^39^	Pakistan	Rural	Three-arm randomized controlled trial	3 months	710 CHWs (710 women, 100%)	Financial

CHW: community health worker.

^a^ More details of the incentives in each study are in Table 2 and the authors’ data repository.^20^

Note: Anganwadi workers are a cadre of CHW in India.

### Study designs

Of the 12 included studies, 11 were randomized controlled trials and one was a non-randomized trial.^31^ Eight studies tested financial performance-based incentives against the usual care (that is, normal working conditions).^28^^,^^30^^–^^35^^,^^39^ One study compared financial and non-financial performance-based incentives separately against usual care.^36^ Three studies tested non-financial performance-based incentives compared with usual care,^37^ an existing intervention^29^ or a video intended to enhance CHWs’ motivation.^38^

The financial performance-based incentives tested included performance-based payments (nine studies),^28^^,^^30^^–^^33^^,^^35^^,^^39^ including income from selling health products, such as diarrhoea treatments.^34^^,^^36^ Non-financial performance-based incentives included the provision of material goods to CHWs, such as household goods and office assets (two studies),^29^^,^^37^ or social recognition, such as a publicly awarded certificate (two studies).^36^^,^^38^ The majority of studies (10 studies) examined the impact of CHW activities on maternal, newborn and child health programmes.^29^^–^^35^^,^^37^^–^^39^ More details of the treatment arms of each study are in the data repository.^20^

### Effects of interventions

**Fig. 3:**
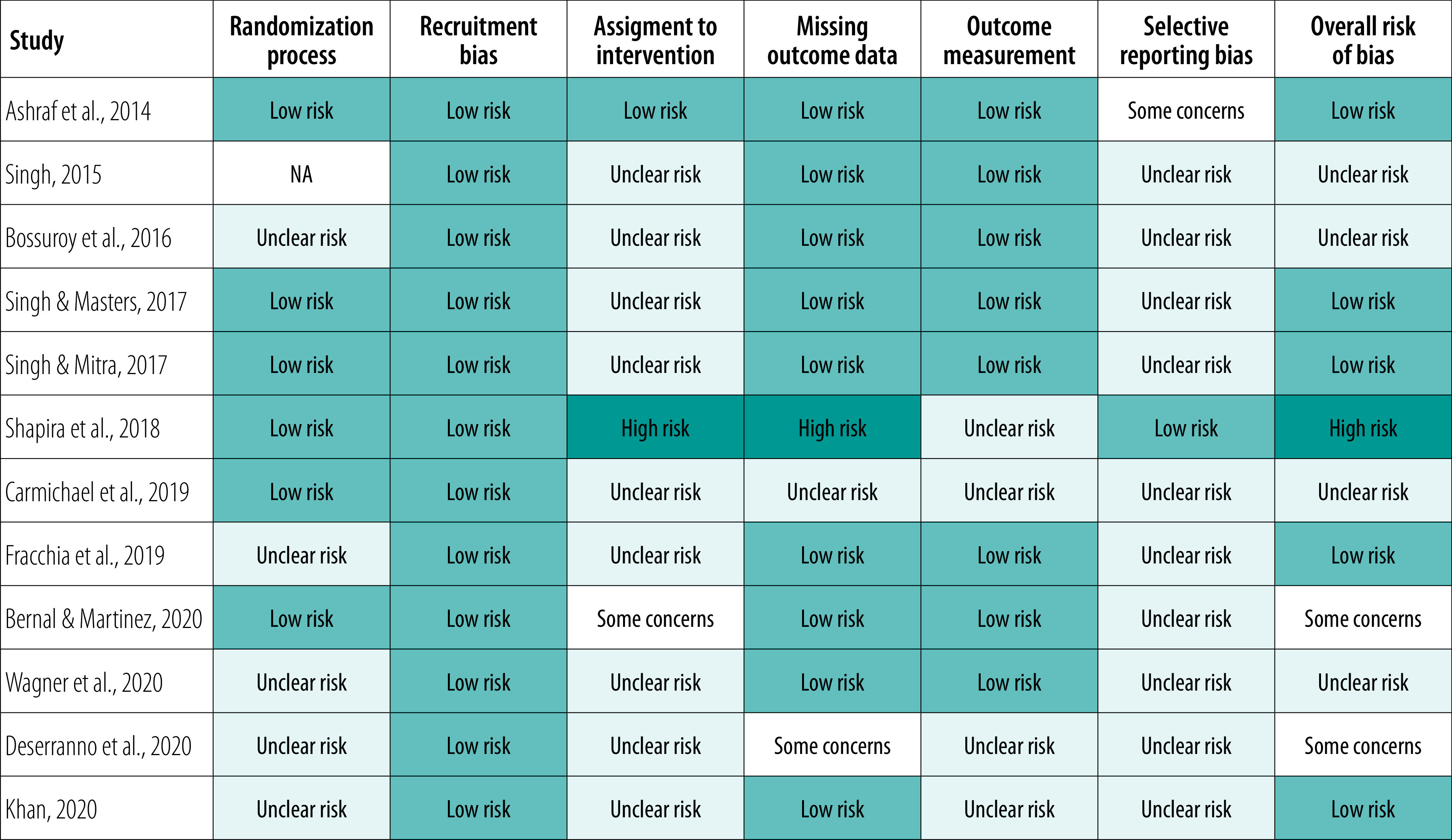
Risk of bias summary for studies included in the systematic review of performance-based incentives for community health workers in low- and middle-income countries

The results of each study are summarized in Table 2. Due to different ways of reporting the data and units of analyses across the papers, we report the results as in the original papers. Only six studies reported their method of randomly allocating participants to groups and several studies relied on subjective outcome measures, primarily household surveys and data collected by CHWs. Results were commonly reported with limited details, particularly regarding baseline data, precision estimates and interpretation. It is therefore unclear whether selective reporting occurred as study protocols were rarely available (Fig. 3); more details are in the data repository.^20^

**Table 2:** Measure of effect for primary outcomes in the systematic review of performance-based incentives for community health workers in low- and middle-income countries

Study & topic	Intervention type	Description of treatment arms	Outcomes (follow-up period)	Measurement	Measure of effect
**CHW competency**					
Ashraf et al., 2014^36^	Financial and non-financial incentives	Intervention A: CHWs keep 90% of retail price for each condom pack sold Intervention B: CHWs keep 10% of retail price for each condom pack sold Intervention C: CHWs publicly display the number of condom packs sold Control: No incentives	CHWs: Number of condom packs sold (12 months)	Number of condom packs each agent restocked over the study period	Intervention A: No statistically significant effect on number of condom packs sold Intervention B: No statistically significant effect on number of condom packs sold Intervention C: 7.48 more condom packs sold (*P* < 0.01) (No further data on treatment effect reported)
Bossuroy et al., 2016^28^	Financial incentive	Intervention A: Financial incentives based on patient detection for 6 months then based on treatment adherence Intervention B: Financial incentives based on patient detection for 6 months then a fixed salary Control: Fixed salary	CHWs: Tuberculosis case detection; CHWs’ motivation Patients: Tuberculosis default rate (6 months)	Administrative data including CHW salary per month CHW surveys	Intervention A: Number of new tuberculosis cases detected increased by 2.18 (33.2%) each month (*P* = 0.01). Over the same period, number of patients defaulting from treatment increased by 0.08 (100%) per month (*P* = 0.05) Intervention B: No statistically significant effect on number of detections or defaults (Baseline information, numerator and denominator not reported)
Carmichael et al., 2019^29^	Non-financial incentive	Intervention: Non-financial incentives for CHW teams if they meet five of seven maternal, newborn, child health indicators per quarter Control: training and monitoring of CHWs on maternal, newborn, child health to increase the quantity and quality of home visits	CHWs: Seven maternal, newborn, child health indicators (2.5 years)	Endline survey of CHWs Baseline and endline surveys of mothers who had given birth in the catchment areas of the intervention and control subcentres	Intervention: Proportion of mothers reporting antenatal household visits increased from 33.3% (277/831) to 64.7% (556/859; *P* = 0.01). Difference-in-difference estimates found that 15% of this difference was attributable to the intervention (*P* = 0.027)
Fracchia et al., 2019^38^	Non-financial incentive	Intervention A. Non-financial prize for CHWs who achieve performance targets Intervention B: CHWs watch a video emphasizing the importance of CHW work Intervention C: Households receive information on the role of CHWs in communities Control: No performance-based incentive	CHWs: Number of household visits; CHWs’ knowledge Patients: Satisfaction with CHWs, self-reported health knowledge (14 months)	Administrative data Baseline and endline CHW surveys Baseline and endline household face-to-face surveys Household endline phone survey	Intervention A: No statistically significant effect on household visits. Household satisfaction with CHWs increased by 0.25 SD (*P* < 0.10). Household knowledge of health practices increased by 0.23 SD (*P* < 0.01) Intervention B: No statistically significant effect on outcomes Intervention C: No statistically significant effect on outcomes
Wagner et al., 2019^34^	Financial incentive	Intervention A: CHWs sell oral rehydration salts and zinc tablets during home visits and retain profits Intervention B: CHWs provide households with vouchers which can be redeemed for oral rehydration salts and zinc tablets Intervention C: CHWs distribute oral rehydration salts and zinc tablets to households free of charge Control: CHWs sell oral rehydration salts in addition to other health products	CHWs: Number of household visits Patients: Use of oral rehydration salts (1 month)	Baseline and endline household surveys	Intervention A: 35% of households visited by CHW Intervention B_:_ 56% of households visited by CHW Intervention C: 61% of households visited by CHW (Baseline information, numerator and denominator not reported)
Bernal et al., 2020^37^	Non-financial incentive	Intervention: Non-financial incentives for community health teams who achieve targets Control: Eligible for incentives after 12 months	CHWs: Community outreach (1.5 years)	Indicators measured every 6 months (including baseline) using household surveys	Intervention: Family planning information provided to women increased from 50.4% to 56.2%, a 5.8% increase compared with control (*P* < 0.10). Knowledge of treatment of diarrhoea among households increased from 7.1% to 15.0%, a 7.8% increase compared with control (*P* < 0.05) (Numerator and denominator not reported)
Deserranno et al., 2020^35^	Financial incentive	Intervention A: CHWs receive performance payments for each household visit conducted Intervention B: Supervisors receive performance payments for each household visit by CHWs under their supervision Intervention C: CHW and supervisors both receive performance payments for each household visit Control: No performance-based incentive	CHWs: Number of household visits; supervisor engagement with community Patients: Self-reported health outcomes (14 months)	Baseline and endline CHW and supervisor surveys Endline household survey	Intervention A: Number of household visits increased by 2.1 (*P* < 0.01) Intervention B: Number of household visits increased by 2.1 (*P* < 0.01) Intervention C: Number of household visits increased by 3.3 (*P* < 0.01) (Baseline data not reported)
Khan, 2020^39^	Financial incentive	Intervention A: CHWs watch a video that emphasizes their mission every 3 months Intervention B: CHWs receive performance payments for every additional household visited (above baseline) Intervention C: both intervention A and B Control: No performance-based incentive	CHWs: Number of household visits Patients: Self-reported health outcomes (3 months)	Baseline and endline household surveys Baseline CHW survey Administrative data	Intervention A: Probability of a household visit was 41.0%, an increase of 5.7% compared with control 35.3% (*P* < 0.01) Intervention B: Probability of a household visit was 45.0%, an increase of 9.7% compared with control 35.3% (*P* < 0.01) Intervention C: Probability of a household visit was 42.1%, an increase of 6.8% compared with control 35.3% (*P* < 0.01) (Baseline, numerators and denominators not reported)
**CHW well-being**					
Bossuroy et al., 2016^28^	Financial incentive	Intervention A: Financial incentives based on patient detection for 6 months then based on treatment adherence Intervention B: Financial incentives based on patient detection for 6 months then a fixed salary Control: Fixed salary	CHWs: Tuberculosis case detection; CHWs’ motivation Patients: Tuberculosis default rate (6 months)	Administrative data including CHW salary per month CHW surveys	Intervention A: CHW job satisfaction decreased by 0.25 SD (*P* < 0.05) Intervention B: CHW job satisfaction decreased by 0.20 SD (*P* < 0.1) (No further data reported)
**Community access**					
Shapira et al., 2018^30^	Financial incentive	Intervention A: CHWs receive performance payments based on the number of targeted maternal, newborn, child health services provided Intervention B: Health centres provide non-financial incentives to mothers who met service utilization indicators Intervention C: both intervention A and B Control: No performance-based incentive	CHWs: Antenatal care visits Patients: Service use (e.g. facility-based deliveries) (3 years)	Baseline and endline household and CHW surveys	Intervention A: Women were 5% less likely to report receiving antenatal care from CHWs (*B* = −0.054; *P* = 0.013). Women were 5% less likely to be referred or accompanied to deliveries (*B* = −0.053; *P* = 0.1) (Numerators and denominators not reported)
**Health outcomes**					
Singh et al., 2015^31^	Financial incentive	Intervention A: Anganwadi workers receive performance payments for each child whose malnutrition classification improved Intervention B: Mothers of children receive a recipe book and Anganwadi workers receive fixed wages Intervention C: both intervention A and B Control: Base salary, no incentives	Patients: Child health outcomes (3 months)	Baseline and endline anthropometric measurements of children Baseline and endline interviews with mothers of children	Intervention A: No statistically significant effect Intervention B: No statistically significant effect Intervention C: Children’s weight increased by 171 g on average compared with control (*P* < 0.05) from a baseline of 12.97 kg. Child malnutrition decreased by 4.2% (from baseline of 43%; *P* < 0.1)
Singh & Masters, 2017^32^	Financial incentive	Intervention A: Anganwadi workers receive performance payment (Rs. 200) for each child whose malnutrition classification improved. Mothers receive a free recipe book Intervention B: Anganwadi workers receive a one-time bonus of Rs. 200 regardless of performance. Mothers receive a free recipe book Control: Base salary, no incentives	Patients: Child health outcomes (3 months)	Baseline and endline anthropometric measurements of children Endline interviews with mothers of children	Intervention A: Children’s weight increased by 219 g on average compared with control (from baseline of 13.67 kg; *P* < 0.01). Child malnutrition decreased by 5.6% (from baseline of 36%; *P* < 0.05) Intervention B: No statistically significant effect
Singh & Mitra, 2017^33^	Financial incentive	Intervention A: Anganwadi workers receive performance payments (Rs.100) for each child whose malnutrition classification improved. Mothers receive a recipe book Intervention B: Same as intervention A but Anganwadi workers receive Rs. 200 per child Intervention C: Anganwadi workers receive performance payments based on their performance relative to others. Mothers receive a free recipe book Control: Base salary, no incentives	Patients: Child health outcomes (3 months)	Baseline and endline anthropometric measurements of children Endline interviews with mothers of children	Intervention A: No statistically significant effect Intervention B: Weight increased by 222 g on average compared with control (*P* < 0.05) from baseline of 13.45 kg. Child malnutrition decreased by 5% (from baseline of 35%; *P* < 0.01) Intervention C: No statistically significant effect

*B*: regression coefficient; CHW: community health worker; CI: confidence interval; Rs: Indian rupees; SD: standard deviation.

^a^ More details of the incentives and the treatment arms in each study are in the authors’ data repository.^20^

NA: not applicable.

Note: More details of the risk of bias and the quality assessment results are in the authors’ data repository.^20^

Studies reported on the following performance outputs of the CHW performance measurement framework:^26^ (i) CHW competency; (ii) CHW well-being; (iii) community access; and (iv) health outcomes. No studies reported on the quality of care provided by CHWs or on community-centred care.

#### CHW competency

Eight studies reported the impact of performance-based incentives on CHW service delivery.

Three studies examined the impact of non-financial performance-based incentives alone on CHW activity levels.^29^^,^^37^^,^^38^ In El Salvador and India, the best performing CHW teams were rewarded with non-financial incentives at a public function (household goods and office equipment).^29^^,^^37^ In India, mothers in the catchment area of the intervention group reported a 15% increase in the number of antenatal home visits by CHWs (*P* = 0.03; baseline numbers not reported).^29^ In El Salvador, family planning information provided to women aged 15–49 years increased by 5.8% (from 50.4% to 56.2%) compared with the control (*P* < 0.10). Householders’ knowledge about treatment of diarrhoea increased by 7.9% (from 7.1% to 15.0%) compared with the control (*P* < 0.05). Both indicators were taken as a proxy measure of increased community outreach by CHWs.^37^ In Guinea-Bissau, when CHWs received a social status award for good performance (a certificate awarded in a public ceremony) householders’ satisfaction with CHWs increased by 0.25 standard deviation (SD) units (*P* < 0.10) and householders’ knowledge of health practices increased by 0.23 SD units (*P* < 0.01; baseline numbers not reported).^38^

Only one study compared the impact of financial and non-financial performance-based incentives on CHWs’ activity levels.^36^ In Zambia, community health agents were recruited to sell packs of condoms. CHWs in the two financial incentive groups received payments in terms of a small (10%) or large (90%) margin of the retail price of each pack sold, while those in the non-financial group received a display that showed to the public the number of packs sold. Neither of the financial performance-based incentives affected CHWs’ performance. However, CHWs in the non-financial incentive group sold 7.48 more packs over the study period: over twice as many packs as CHWs in the control group with no incentives (*P* < 0.01). 

Four studies examined the impact of financial performance-based incentives alone on CHWs’ activity levels.^28^^,^^34^^,^^35^^,^^39^ In Sierra Leone, CHWs receiving financial performance-based incentives made an additional 2.1 household visits on average (*P* < 0.01) over 6 months.^35^ When both CHWs and their supervisor received financial incentives, CHWs made 3.3 more visits on average (*P* < 0.01; baseline numbers not reported). In India, when CHWs had financial incentives to detect tuberculosis cases, the number of new cases detected increased by 2.18 (33.2%) on average each month over 6 months (*P* < 0.05).^28^ However, the number of patients defaulting from treatment also increased in the intervention group during this period by an average of 0.08 per month (100%; *P* < 0.05; baseline values not reported). In Pakistan, CHWs received 25 Pakistani rupees for every additional household they visited.^39^ In the intervention group the probability of a household visit by CHWs was 9.7% higher than the control group probability (45.0% versus 35.3%; *P* < 0.01). Lastly in Uganda, CHWs who retained the profits from selling oral rehydration salts and zinc tablets visited 35% of households in their catchment area, compared with 61% of households among CHWs who distributed these products for free.^34^

#### CHW well-being

One study used surveys to assess the impact of performance-based incentives on CHWs’ job satisfaction in India.^28^ The researchers reported that when CHWs had financial incentives to detect tuberculosis cases their job satisfaction decreased by 0.25 SD units and when they were incentivized to prevent defaults from treatment, job satisfaction decreased by 0.20 SD units (before-and-after measures not reported). Qualitative interviews suggested that dissatisfaction among CHWs was directed largely at incentives to prevent patients defaulting, over which CHWs felt they had little control, resulting in lower marginal returns on effort.

#### Community access

One study measured the impact of financial performance-based incentives for CHW cooperatives in Rwanda on the utilization of health services by their communities.^30^ Financial incentives appeared to have no impact on coverage of the targeted services or on number of hours spent providing health services each week by CHWs. Mothers in villages where CHWs received performance payments alone were 5% less likely to receive antenatal care advice (*P* = 0.013; baseline numbers not reported).

#### Health outcomes

Three studies measured the impact of performance-based incentives on health outcomes.^31^^–^^33^ The studies were all conducted in child day-care facilities in urban slums in India, where Anganwadi workers received performance payments based on improvements in children’s malnutrition. The first study identified a 4.2% decline in weight-for-age malnutrition (from 43.0% to 38.8%; *P* < 0.01) over 3 months when Anganwadi workers received 100 Indian rupees (Rs.; United States dollars, US$ 1.4) for each child whose weight improved and when the mothers received a free recipe book.^31^ However, when the financial incentive and recipe book were tested individually, the effects were negligible. A follow-up study found that a similar low payment (Rs. 100; US$ 1.4) to Anganwadi workers had no significant effects on children’s malnutrition compared with a higher payment (Rs. 200; US$ 2.7), which reduced malnutrition by 5.3% over 3 months (*P* < 0.05).^33^ Lastly, a third study of performance-based incentives for Anganwadi workers (Rs. 200; US$ 2.7 for each child with improved weight) found a 5.6% reduced prevalence of malnutrition on average over 3 months (from 36% to 31%).^32^

### Pathways to impact

Eight of the 12 included studies explored the mechanisms behind the impact of the performance-based incentives studied. Table 3 provides a summary of what did and did not work in terms of the design of performance-based incentives in the included studies.

**Table 3:** Summary of evidence of effectiveness of types of performance-based incentives for community health workers in low- and middle-income countries

Type of performance-based incentive	Mechanism of incentive	Why incentive worked	Countries
**What incentives worked?**			
Financial	Providing performance payments to both CHWs and their supervisors	Complementary efforts from CHWs to supply services and from supervisors raised demand for services^35^	Sierra Leone
	Pairing performance payments with complementary demand-side information	Incentives for Anganwadi workers and information for mothers resulted in better communication and the incentivized workers made more home visits to monitor healthy cooking^31^^–^^33^	India
Non-financial	Team-based goals and targets	Incentives supported intrinsic motivation and team cohesion^29^^,^^37^	El Salvador, India
	Boosting social status of CHWs by awarding certificates and rewards in public ceremonies and meetings	Incentives can reinforce intrinsic motivation of CHWs and families’ support of CHWs^29^^,^^38^ Incentives can promote community recognition and appreciation of CHWs^37^	El Salvador, Guinea-Bissau, India
	Facilitating peer comparison among CHW	Incentives can encourage effort or help CHWs to assess what is expected of them^36^	India, Zambia
**What incentives did not work?**			
Financial	Small performance payments to CHWs	On aggregate, low financial incentives were not sufficient to motivate additional effort by CHWs^30^^,^^36^	Rwanda, Zambia
	Limited control over incentivized tasks	Incentivized tasks perceived to be outside the control of CHWs reduced their effort and may have led to unrewarded tasks being neglected^28^ For CHW cooperatives, performance depended on the unobservable efforts of the other cooperative members^30^	India, Rwanda
	Complex rules around the disbursement of performance-based incentives	CHWs reported confusion about the payment mechanisms (which were intended to encourage team and individual effort)^30^	Rwanda
	Selling products to known impoverished households	CHWs reported feeling embarrassed and socially penalized, which resulted in lower effort^34^	Uganda
	Providing performance payments for a subset of CHW activities	CHWs reallocated their effort towards the rewarded task to the detriment of other unrewarded activities^28^^,^^39^ CHWs focused their effort on supporting community members closest to the target measure, at the expense of other tasks^32^^,^^33^	India, Pakistan

CHW: community health worker.

Four studies attributed the positive impact of non-financial performance-based incentives to an increase in the social status of CHWs.^29^^,^^36^^–^^38^ In Guinea-Bissau, improved performance was measured among CHWs awarded with a prize during a ceremony with the presence of health authorities and community members.^38^ In Zambia, non-financial incentives were more effective when CHWs had more peers in the same neighbourhood and they reported being motivated by showing off their own sales levels and viewing the sales levels of their peers.^36^

The prosocial motivation of CHWs (the desire to act for the benefit of others or with the intention of helping others) was only measured in one study. In Zambia, CHWs were invited to donate to an existing charity that provided care to patients with human immunodeficiency virus infection or acquired immunodeficiency syndrome.^36^ CHWs who donated more were considered to have a higher level of prosocial motivation. Both financial and non-financial performance-based incentives were found to be more effective for those with a high level of prosocial motivation, as CHWs sold 51% more condoms than the average agent in the control group over 12 months (baseline not reported).

Six studies found that the amount of a financial performance-based incentive influenced the incentive’s impact (one study tested both high- and low-value performance payments).^30^^–^^33^^,^^35^^,^^36^ Low financial incentives were insufficient to elicit additional effort from CHWs in four studies.^30^^,^^31^^,^^33^^,^^36^ In Rwanda, for example, financial incentives for CHW cooperatives were originally set at US$ 2.1–3.2 per service but reduced to US$ 0.5–1.0 by year 2 due to budgetary constraints. Conversely, high performance payments were found to be effective in three studies.^32^^,^^33^^,^^35^ In Sierra Leone, for instance, CHWs could earn up to an additional 40% of their monthly wage of 150 000 Sierra Leonean Leone (US$ 19.5 at the time of the study) in financial incentives.^35^

Four studies of financial performance-based incentives found evidence of individuals allocating their effort towards incentivized tasks or outcomes while neglecting non-contracted activities.^28^^,^^32^^,^^33^^,^^39^ In India, Anganwadi workers reallocated their effort towards the incentivized activity (detecting new cases of tuberculosis), to the detriment of other non-rewarded tasks.^28^ Similarly, Anganwadi workers allocated their effort to children who were closest to the malnutrition cut-off threshold (that is, those providing the highest financial returns on effort).^32^^,^^33^ Improvements in health outcomes were almost 70% higher for children closest to their target weight compared with those further away (baseline not reported).^33^

## Discussion

We found that non-financial performance-based incentives were consistently effective at increasing CHW service delivery outcomes. While large financial incentives alone were more effective than small ones, they occasionally resulted in unintended consequences such as CHWs reallocating effort towards incentivized activities to the detriment of unrewarded tasks. Additionally, we did not find any studies testing a combination of financial and non-financial incentives.

Our findings reflect the recommendations of the 2018 WHO guideline on CHW programmes that while incentives are clearly important for CHWs, there is a need for caution with the exclusive use of financial performance-based incentives.^13^ Although the studies included in this review did not always report whether CHWs received baseline forms of remuneration, seven out of nine studies on financial incentives provided incentives on top of baseline remuneration.^28^^,^^31^^–^^33^^,^^35^^,^^36^^,^^39^ In the two studies providing performance-based financial incentives alone,^30^^,^^34^ the incentives were detrimental to CHW performance. In four out of seven studies where baseline remuneration was present, CHWs were found to allocate effort towards incentivized indicators at the expense of other, non-incentivized tasks.^28^^,^^32^^,^^33^^,^^39^ These findings align with the theory that incentivizing a subset of tasks or outcomes can lead to a reduction in effort devoted to non-contracted outcomes.^18^ Thus, despite limited information about the context of the studies in our review, it seems that financial incentives need to be approached with caution, regardless of baseline remuneration.

Financial performance-based incentives that showed signs of working were underpinned by a small number of common mechanisms. These included the use of complementary strategies. For instance, in Sierra Leone, giving incentives to supervisors of CHWs stimulated them to boost demand for CHW services.^35^ Similarly, in India, Anganwadi workers conducted more household visits, while mothers received free recipe books to cook more nutritious food at home.^31^^–^^33^ These findings may demonstrate that complementing financial incentives with other sources of motivation of CHWs, such as their supervision network or the community they serve, may further enhance performance.^40^

Our finding that the size of financial performance-based incentives influenced CHW performance is consistent with existing evidence.^41^ Large financial incentives may convey positive information to CHWs about the value of their work, resulting in higher effort, while small incentives appear to lack the incentive effect necessary to influence behaviour and are commonly reported as a source of demotivation for CHWs.^42^^–^^44^ However, larger performance-based incentives may also detract CHWs’ attention from unrewarded activities.^33^^,^^45^ Additionally, whether the impact of large incentives endures beyond the short-term was not assessed by any of the studies included in this review.^6^

The mechanisms underpinning the positive impact of non-financial performance-based incentives on CHW performance appear more direct. This type of incentive seems to promote both intrinsic motivation and social recognition (also a well-documented source of motivation).^7^^,^^46^ Indeed, two studies conducted subsequent qualitative research in which CHWs reported that the enhanced social status from receiving non-financial incentives in public was more motivating than the incentives themselves.^47^^,^^48^ This finding may be of particular relevance to volunteer CHW programmes, where performance payments may be more likely to undermine intrinsic motivation.^43^^,^^49^^,^^50^ However, recent evidence demonstrates that the design of non-financial incentives is not always straightforward. Rewards may be detrimental to performance if they are not perceived to be fair and objective, or frequent enough to motivate effort.^51^ Additionally, rewards must be designed with the local context in mind, including how they will address the expectations and motivations of CHWs.^40^^,^^49^^,^^52^

Importantly, these findings should not be taken to mean that non-financial performance-based incentives can be seen as a substitute for financial remuneration (and vice versa).^13^ The need for a mix of incentives to support CHWs is well-documented.^4^^,^^10^^,^^53^ However, despite recommendations dating back approximately two decades, we did not find any studies that tested a package of incentives.^21^^,^^48^ Only two of the included studies acknowledged the importance of a mix of incentives, but opted to test non-financial incentives due to concerns about the sustainability of financial incentives.^29^^,^^38^ As countries move to professionalize CHW programmes with appropriate forms of remuneration, research addressing this gap will be increasingly important.

Our review is limited in three main ways. First, other study designs (for example, qualitative) may be more appropriate than intervention studies to capture the complex systems within which incentive schemes are introduced. However, our primary focus was to assess the impact of performance-based incentives.^54^ This focus may explain why the included studies only partially covered the domains of the CHW performance measurement framework. Second, inconsistent study design precluded a meta-analysis. Lastly, some of the studies included in our review implemented interventions simultaneously with other quality improvement initiatives and it is possible that the impact of the incentive itself was overestimated.

In conclusion, non-financial incentives can be effective at increasing CHW motivation and performance when the incentives promote social recognition. Financial incentives alone can improve CHW service delivery outcomes, but at the risk of unincentivized tasks becoming neglected. The absence of research on a combined package of incentives is notable, particularly as resource-poor countries face challenges to professionalize their CHW programmes with appropriate packages of remuneration. Future research must pay greater attention to the design, context and sustainability of incentives.
